# Editorial: Targeting Protein Post-Translational Modifications (PTMs) for Diagnosis and Treatment of Sepsis

**DOI:** 10.3389/fimmu.2022.856146

**Published:** 2022-02-03

**Authors:** Panpan Chang, Yongqing Li

**Affiliations:** ^1^ Trauma Medicine Center, Peking University People’s Hospital, Beijing, China; ^2^ Key Laboratory of Trauma and Neural Regeneration, Peking University, Beijing, China; ^3^ National Center for Trauma Medicine of China, Beijing, China; ^4^ Department of Surgery, University of Michigan Medical School, Ann Arbor, MI, United States

**Keywords:** protein posttranslational modifications (PTMs), sepsis, biomarker, treatment, diagnosis

Sepsis is a serious clinical problem that is associated with unacceptably high mortality and for many of those who survive long-term morbidity (1). Although accumulating evidence and studies have increased understanding of this problem in the past 10 years, it remains lack of effective diagnosis and treatment to improve the outcome due to its complicated pathophysiological mechanisms. Growing evidence suggests that post-translational modifications (PTMs) could be a cornerstone in regulating cell functions and multiples diseases, including sepsis. It is unclear exactly how infection induces specific protein PTMs, and how the protein PTMs consequently lead to inflammatory disorder, cytokine storm, disseminated intravascular coagulation, organ dysfunction, *etc.* This Research Topic of *Frontiers in Immunology* introduced latest progress in modulation of PTMs in infection and immune response, PTMs-induced transcriptional reprogramming of immune cells in severe inflammation and/or infection, and proteins derived from PTMs as sepsis diagnostic biomarkers and therapeutic targets (**Figure 1**).

**Figure 1:**
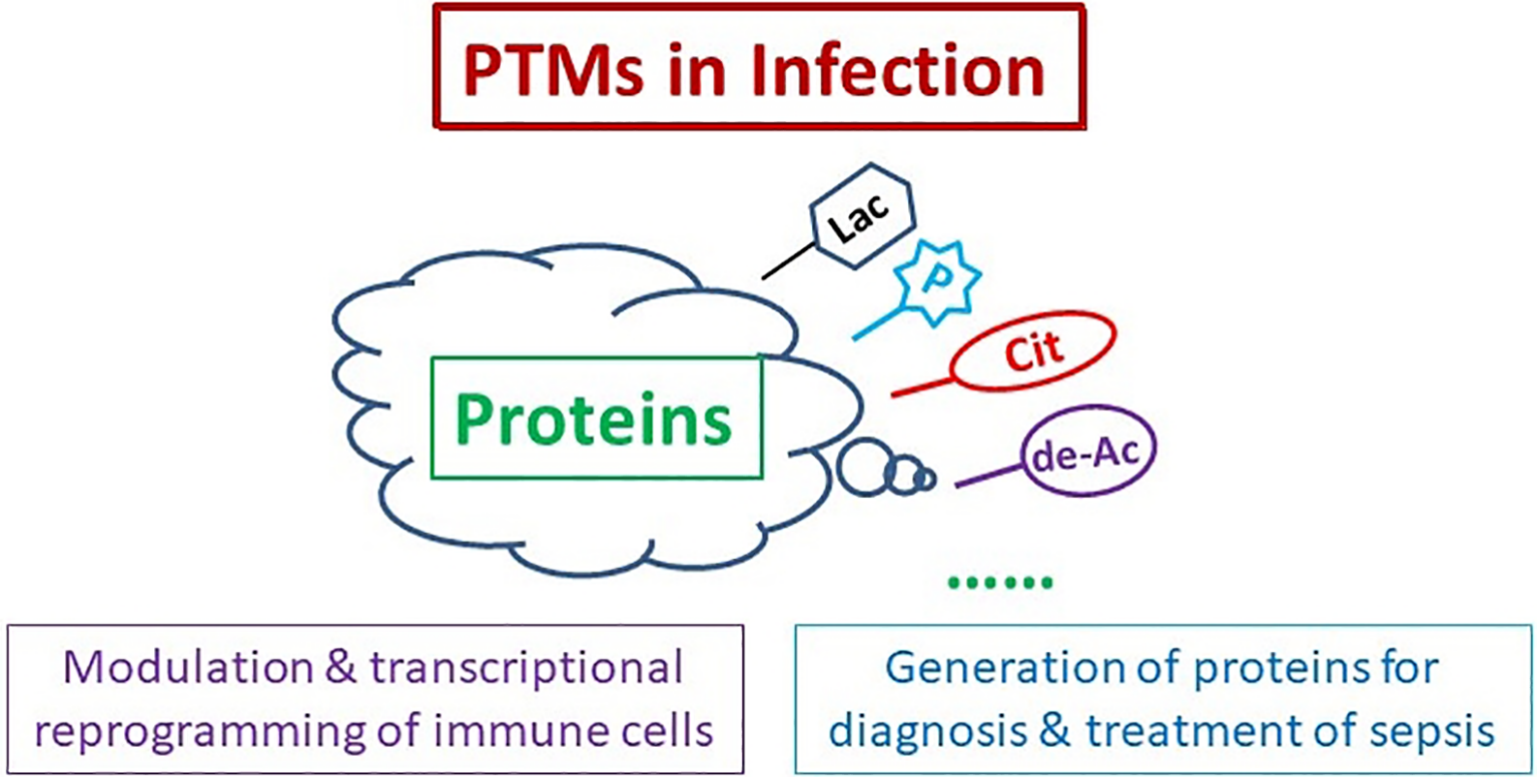
Protein Post-translational Modifications (PTMs) for Diagnosis and Treatment of Sepsis. PTMs of proteins participate in modulation and transcriptional reprogramming of immune cells, and generation of the proteins for sepsis diagnosis and treatment. Cit, citrullination; de-Ac, deacetylation; Lac, lactylation; P, phosphorylation.

## Modulation of PTMs in Infection and Immune Response

The findings by Sun et al. described that deacetylation of p53 promotes autophagy of renal tubular epithelia cells (RTEC) to alleviate sepsis-induced acute kidney injury (AKI). The p53 could be regulated by various PTMs such as phosphorylation, acetylation, methylation, glycosylation (2). Emerging evidence indicates that autophagy plays protective roles in various types of AKI (3), and upregulation of p53 could mediate the attenuation of autophagy in AKI (4). The study by Sun et al. emphasizes the potential of deacetylated p53-mediated RTEC autophagy for future sepsis-induces AKI treatments. Zhao et al. updated the role of high mobility group box protein 1 (HMGB1) in disrupting the endothelial barrier integrity during acute lung injury (ALI) in sepsis. This study demonstrates that HMGB1 induced F-actin rearrangement, adherens junction and tight junction rupture lead to increase of the endothelial barrier permeability through the RAGE/ROCK1 pathway, which phosphorylates myosin light chain in the early stage. As increased vascular permeability is essential to pulmonary edema during sepsis-induced ALI (5), this finding confirmed a potential therapeutic target for the endothelial cell (EC) barrier dysfunction following sepsis. Yang et al. revealed that miR-221-5p downregulated c-Jun N-terminal protein kinase 2 (JNK2) to aggravate sepsis-induced ALI. This team has previously demonstrated that JNK2 promotes stress-induced mitophagy by targeting small mitochondrial alternative reading frame (smARF) for ubiquitin-mediated proteasomal degradation, thereby preventing mitochondrial dysfunction and restraining inflammasome activation (6). The present study has advanced the field further by providing a potential mechanism for the association between mitochondrial dysfunction and sepsis. Chang et al. summarized histone deacetylase 6 (HDAC6) regulating autophagy and NLRP3 inflammasomes *via* multiple mechanisms. HDAC6 has two functional deacetylase domains and a ubiquitin-binding zinc finger domain (ZnF-BUZ) (7). Due to its unique structure, HDAC6 regulates various physiological processes, including autophagy and NLRP3 inflammasome. With the development of small molecules inhibiting HDAC6, some clinical trials have shown that selective HDAC6 inhibitors are effective in tumor treatment (8–10). Considering the function of HDAC6 in autophagy and NLRP3 inflammasome and broad role of HDAC6 inhibitors, further studies should be pursued in future research.

## PTMs-Induced Transcriptional Reprogramming of Immune Cells in Severe Inflammation and/or Infection

Wu et al. discussed the role of peptidylarginine deiminase 2 (PAD2) in host immunity. PAD family enzymes catalyze the conversion of arginine residues to citrulline, regulating activity of host immunity. PAD2 mediates protein citrullination to regulate multiple cellular processes including gene transcription (11), antigen generation (12), extracellular trap formation (13), and pyroptosis (14). Dysregulated activity of PAD2 is associated with a series of immune disorders including sepsis, rheumatoid arthritis, multiple sclerosis, and tumor formation. Recently, Li’s group found that PAD2 mediates pyroptosis and neutrophil extracellular traps (NETs) during sepsis (15, 16). Accumulating evidence suggests that PAD2 may be a promising novel biomarker and therapeutic target for a broad spectrum of diseases such as sepsis, autoimmune and inflammatory diseases. Miao et al. discussed the development of proteomics in sepsis. They summarize the application of proteomics in elucidating the molecular mechanism and potential therapeutic targets of sepsis and the research progress of protein PTMs in sepsis.

## Proteins Derived From PTMs for Sepsis Diagnosis and Treatment

In 2011, Li’s group first identified citrullinated histone H3 (CitH3) as a biomarker for early diagnosis and prognosis of endotoxic shock in a mouse model (17). In next series of studies, they developed a novel CitH3 monoclonal antibody to target PAD2 and PAD4 generated CitH3 (18) and confirmed that CitH3 is more effective than existing biomarkers such as procalcitonin (PCT), interleukin (IL) 1β, and IL-6 in a mouse model of endotoxic shock and human patients with sepsis (19, 20). However, the mechanisms involved are unveiled. In this Research Topic, CitH3 is further discussed in three manuscripts. Wu et al. showed that activation of PAD enzymes by microbial infection induces formation of NETs and pyroptosis, leading to CitH3 released from immune cells such as neutrophils and microphages. Tian et al. further revealed that CitH3 activates caspase-1 dependent inflammasome pathway. Their studies illustrate a novel mechanism, suggesting that CitH3 is an important self-mediator that triggers formation a “*vicious circle*” during sepsis and sepsis-induced ALI. Another study from Pan et al. explored the diagnostic potential of CitH3 in septic patients with acute pancreatitis (AP). Their findings indicate that CitH3 concentration is increased in septic AP patients and is closely correlated with disease severity and clinical outcomes. These two groups’ data suggest that CitH3 could be a promising therapeutic target and biomarker in sepsis. Interestingly, Chu et al. reported lactylation of histone H3 lysine 18 (H3K18) in patients with septic shock. H3K9 lactylation has been recently studied in macrophage following hypoxia, lipopolysaccharides, or bacterial infection (21). The data presented by Chu et al. indicate that H3K18 lactylation may be a potential biomarker to reflect the severity of critical illness and the presence of infection. The studies with a large sample size are needed to confirm the conclusion.

Zhai et al. reported that combination of soluble interleukin-2 receptor (sIL-2R), tumor necrosis factor-a (TNF-a) and PCT has good value in the diagnosis of sepsis infection in patients with closed abdominal injury complicated with severe multiple abdominal injuries. Additionally, Wang et al. showed G-protein coupled receptor 174 (GPR174) mRNA acts as a novel prognostic biomarker for patients with sepsis. GPR174 is involved in the dysregulated immune response of sepsis, however, the clinical value and effects of GPR174 in septic patients are still unknown. Wang et al.’s study indicates the role of GPR174 regulating inflammation following sepsis and suggest the potential of GPR174 as a prognosis biomarker for sepsis.

An important goal of this Research Topic was to highlight the scientific community of advances made on the discovery of new mechanisms from PTMs, to solve the clinical problem, sepsis. The contributed papers serve to illustrate a significant underlying message that various PTMs mediate infection and immune response, regulate transcriptional reprogramming of immune cells in severe inflammation, and work as potential biomarkers for diagnosis and prognosis of sepsis. Further studies are still needed to explore underlying mechanisms and clinical application using a plethora of technologies including bioinformatics, molecular biology studies and rigorous statistical methods.

## Author Contributions

PC and YL wrote the manuscript. YL made critical revision. All authors contributed to the article and approved the submitted version.

## Funding

This work was funded by grants from the National Institute of Health R01 (R01HL155116) to YL, the Joint-of- Institute (Grant# U068874) to YL, National Natural Science Foundation of China (82102315) to PC, and Beijing Natural Science Foundation (7214265) to PC.

## Conflict of Interest

The authors declare that the research was conducted in the absence of any commercial or financial relationships that could be construed as a potential conflict of interest.

## Publisher’s Note

All claims expressed in this article are solely those of the authors and do not necessarily represent those of their affiliated organizations, or those of the publisher, the editors and the reviewers. Any product that may be evaluated in this article, or claim that may be made by its manufacturer, is not guaranteed or endorsed by the publisher.
